# Four propositions on integrated sustainability: toward a theoretical framework to understand the environment, peace, and sustainability nexus

**DOI:** 10.1007/s11625-021-00925-y

**Published:** 2021-03-10

**Authors:** Joshua Fisher, Poonam Arora, Siqi Chen, Sophia Rhee, Tempest Blaine, Dahlia Simangan

**Affiliations:** 1 The Advanced Consortium on Cooperation, Conflict and Complexity (AC4), Earth Institute, Columbia University, 475 Riverside Drive, 253 Interchurch Center, New York, NY 10115 USA; 2 O’Malley School of Business, Manhattan College, New York, USA; 3 Network for Education and Research on Peace and Sustainability, Hiroshima University, Hiroshima, Japan

**Keywords:** Sustainable development, Integrated sustainability, Theoretical framework, Model development

## Abstract

The sustainability agenda has evolved around a set of interconnected dilemmas regarding economic, social, and environmental goals. Progress has been made in establishing thresholds and targets that must be achieved to enable life to continue to thrive on the planet. However, much work remains to be done in articulating coherent theoretical frameworks that adequately describe the mechanisms through which sustainability outcomes are achieved. This paper reviews core concepts in the sustainability agenda to develop four propositions on integrated sustainability that collectively describe the underlying mechanisms of sustainable development. We then advance a framework for integrated sustainability and assess its viability through linear regression and principal components analysis of key selected indicators. The results provide preliminary evidence that countries with institutions that enable cooperation and regulate competition perform better in attaining integrated sustainability indicators. Our findings suggest that institutional design is important to sustainability outcomes and that further research into process-oriented mechanisms and institutional characteristics can yield substantial dividends in enabling effective sustainability policy.

**Supplementary Information:**

The online version contains supplementary material available at 10.1007/s11625-021-00925-y.

## Introduction

The sustainability and sustainable development agendas have evolved around sets of interconnected dilemmas involving provision of basic standards of living, enabling economic growth, maintaining environmental integrity, and the effective governance of both social and ecological systems. Scientific understanding of the world’s sustainability challenges has become increasingly sophisticated and nuanced with research and policy communities emphasizing the outcomes that must be achieved for life on the planet to continue to thrive. However, any sustainable development agenda is inherently value-laden and thus political (Halla and Binder 2020). While there is broad global consensus around the aspirational set of goals included in the 2030 Agenda for Sustainable Development (UN General Assembly 2015), there is continued disagreement around the appropriate and effective strategies to achieve the Sustainable Development Goals (SDGs). Essentially, throughout the evolution of the 2030 Agenda the world has learned a great deal about what we need to achieve, yet we remain divided regarding how to effectively and equitably balance competing and perhaps incompatible interests among diverse stakeholders (Liebovitch et al. 2020; Razavi 2016).

The SDGs represent an interconnected and integrated approach to development and sustainability. While integration across goals is implicit in the 2030 Agenda, the links across the environmental, economic, and social dimensions of the goals are not explicit enough to strengthen policy integration (Le Blanc 2015) and the agenda relies on assumptions regarding the linkages and interactions across the goals. Such limited elaboration of interconnectivity is particularly acute in goals related to the climate, land, energy and water nexus, between energy and industrialization, and between oceans and climate change (Le Blanc 2015). Social and ecological justice are also narrowly understood in the agenda and limited to redress and access (Dryzek and Pickering 2019). Likewise, although the 2030 Agenda recognizes peace as imperative to sustainable development and vice versa, peace is conceptually and operationally vague and primarily output oriented.

With countries adopting divergent interpretations and approaches to national-level implementation of the goals (Tosun and Leininger 2017), the 2030 Agenda may be impeded by conflicting—and at times incompatible approaches to SDG implementation. The resulting dilemmas are more visible than ever as the world grapples with the myriad challenges involved in the COVID-19 pandemic. The pandemic has exposed deep value differences within and across societies [for examples see Ling and Ho (2020) and Memish et al. (2020)] around such questions as how to protect human health, how to maintain and re-start national and regional economies, what sectors of an economy, and which service providers are ‘essential’ and thus expected to assume higher risk activities, and associated questions of justice and equity in vulnerability and treatment.

Undoubtedly, the pandemic has eroded economic and social capital (Polyakova et al. 2020), although the true magnitude will be unclear until after, when the full extent of human exposure can be quantified (Birkmann 2006). What is already evident is the role of social inequality in both the susceptibility to and the ability to cope with the pandemic, raising questions about the breakdown in the social contract between different groups and the state, in line with expectations articulated in earlier frameworks (Pelling and Dill 2010; Birkmann et al. 2013). Such contested social contracts are not unusual in politicized disasters, especially when relations between citizen groups and the state are unequal. Strong partnerships between community-based organizations, non-governmental and governmental organizations, public and private sectors, and local, regional, and national government are fundamental to rebuilding capital in the short-run and countering deep inequalities in the long-run. Similar dilemmas, inequalities, and conflicts permeate the sustainability agenda, which incorporates competing values around climate change to energy policy to supply chains and business practices, among many others. With such deep divides around existential dilemmas, a more integrated framework becomes crucial for advancing viable policy coherence that can enable citizens and societies to thrive without impeding that same ability for others and without perpetuating fundamental social inequalities and inequities.

Building upon these ideas*,* this paper develops a framework for integrated sustainability, situated in the environment, peace and sustainability nexus to highlight the mechanisms under which societies can better resolve sustainability dilemmas. We begin by discussing the core elements of the sustainability agenda and some of its key dilemmas. They are organized into four propositions and a resulting framework is suggested for integrated sustainability, emphasizing the central role that cooperation and regulated competition play in resolving sustainability dilemmas (Deutsch 2006). Next, we identify a set of proxy measures for the framework and conduct quantitative assessment of the theoretical hypotheses using linear regressions and principal component analysis. The paper then ends with calls for further empirical research to critically evaluate and measure our framework’s components with higher resolution data and more precise models in order to contribute inputs and guidance for evidence-based policymaking.

## The need for a model of integrated sustainability

We argue that at their essence, environmental sustainability and sustainable development discussions (henceforth the ‘sustainability agenda’) consider the question of how societies can optimize human well-being and social and economic life within planetary boundaries (Boyer et al., 2016; Gerten et al. 2020; Rockström et al. 2009), both now and into the future. In pursuit of this agenda, researchers and policymakers have developed increasingly complex insights into the feedback processes and interdependencies that shape various aspects of the world’s social-ecological systems (Jacobi et al. 2020), which have been essential to defining the targets that need to be reached and setting clear goals to mobilize collective action. For instance, the Paris Agreement (2015) provides clear political commitments to emissions reductions related to the climate goals of the 2030 Agenda (UN 2015). Despite such movements towards committed science and policy, according to a UN statement, “the global landscape for Sustainable Development Goal (SDG) implementation has generally deteriorated since 2015”, which makes efforts to leverage integrated sustainability ever more urgent (UN 2019). Of particular importance is how outcomes in the sustainability agenda are framed, whether through targets or institutions themselves. While certain SDGs have existing rules and governance arrangements at international and national levels (i.e., UNFCCC for climate change), others do not. This means that some goals are target-based, leaving questions of governance and operational cooperation mired in difficulties and fragmentation (Kanie et al. 2019). In fact, previous research has shown how goal-based implementation illuminates deep divides along disciplinary, race, class, cultural, political, and paradigmatic lines (Evans and Musvipwa 2017).

In contrast, an effective governance approach requires creating normative coherence and systems-awareness (Biermann et al. 2012), which is often difficult to achieve in practice with multiple stakeholders. Given the number of actors at various scales of social and spatial aggregation with distinct needs and goals, as well as the real limits of the world’s resources and biophysical and geochemical thresholds, there is no optimal or pareto optimal solution to achieving a social-ecological-economic balance (Fisher 2014; Hoberg and Strunz 2018). Instead, we are confronted with the reality that various needs and interests are often practically incompatible, with the implication that any sustainability target or policy necessarily involves making tradeoffs that privilege certain actors’ agendas and values (intentionally or tacitly) over others (Allen et al. 2020; Fisher and Rucki 2017). This can create a class of policy conflicts known as wicked problems (Rittel and Webber 1973). These problems are defined by multiple, interconnected issues and stakeholders, each of whom define the problem uniquely, and hold their own perspectives on what should be done to remedy the issue accordingly. This means that rather than a single problem that the stakeholders commonly confront, there are potentially at least as many problems in an environmental issue as there are stakeholders.

This is further complicated by a dominant worldview which has created a historical division between social and environmental relations. This human-nature dualism has rendered invisible the patterns of ecological destruction embedded in a capitalist world ecology (Moore 2016). At the same time, the logic of capitalist processes makes subjugation and commodification of nature necessary for capital accumulation, in turn creating systemic inequalities (Harvey 2005). Importantly, these inequalities are perpetuated both among human beings, as well as among species and environments. By viewing human–nature relations as a complex system, its interdependencies become apparent (Arora et al. 2016), as well as the ways in which the distributional dimensions of environmental impacts and harms are born differently by groups within societies.

Thus, the pursuit of the sustainability agenda requires constant processes of managing the conflicts that result from incompatible needs, interests, goals, and the boundaries of our biophysical and geochemical systems, as well as the resulting inequalities. Many authors have emphasized the role of integrated implementation, linking sectors and actors across geographies through integrated plans and shared trust (Stafford-Smith et al. 2017; Allen et al. 2020). Previous calls for improving effective environmental action have noted the importance of institutional change at the global level (Biermann et al. 2012). Similarly, in this paper we propose to investigate the processes, structures, and institutions that enable us to effectively manage the dilemmas inherent to the sustainability agenda (Fisher and Coleman 2019). We understand the SDGs themselves as insufficient decision-support instruments, and a deeper study of the complex system and multiple forces that lead to the goals themselves is necessary (Allen et al. 2020). As the majority of the metrics of sustainability are outcome-focused, there is a need to explore and elucidate the mechanisms behind achieving the sustainability agenda (Kozlova et al. 2020; Haque and Ntim 2017; Kovalenko et al. 2016).

While acknowledging the wide variety of human-nature and policy framings around the sustainability agenda, we suggest that by returning to the principal tenets of sustainability, viewed from a complex systems lens and exploring the enabling conditions as well as the underlying processes associated with better sustainability outcomes, it is possible to develop policy guidance to propel the sustainability agenda forward. Where the 2030 Agenda tends to be outcome focused, we complement it by offering insight into the mechanisms that enable integration across its goals.

### Foundations of integrated sustainability

Though many normative framings of sustainability and sustainable development exist (Clune et al. 2020; Kidd 1992), in this paper we approach the terms at their broadest conception. Here, sustainable development can be thought of as the point at which all human beings live in the security that they have the capacity to achieve harmony and self-actualization, both now and into the future (Brundtland 1987). Stedman and Hill (1992, p 1) state that “sustainable development is about human well-being—our utter dependence on natural resources and our almost universal desire for economic improvement”. When interpreting this definition through the lens of complex, interconnected and evolving systems (Fisher and Rucki 2017), we argue that our dependence on natural resources is not merely exploitative and consumption-driven, but also oriented towards human well-being in many forms—not only from ecosystem services but also the intrinsic value of nature on mental health and well-being (Constanza et al. 1997; Basu et al. 2020). The OECD (2001, p 11) extends the conception of well-being, that it is “more than the sum of individual levels of well-being since it relates to individual and societal preferences regarding equality of opportunities, civil liberties, distribution of resources and opportunities for further learning”. Consequently, the sustainability agenda involves the pursuit of certain social characteristics that allow for self-actualization across social aggregations, from individuals to the global collective.

This view is similar to understandings of peace and freedom. Peace is not only the absence of war and direct or somatic violence (i.e., negative peace); it is also the absence of structural and indirect forms of violence (i.e., positive peace), which is part and parcel of achieving well-being (Galtung 1964). Positive peace is manifested in social harmony and cooperation, consisting of freedom from fear, freedom from want, economic growth and development, absence of exploitation, and equality, among other factors (Galtung 1969). This holistic definition of peace includes freedom from less visible forms of violence, such as social discrimination, political censorship, and other structural inequities that prevent the flourishing of individual agency and opportunity. Along the same lines, freedom is more than just the absence of restraints (i.e., negative freedom), it is also the presence of conditions that enable a person to achieve certain aspirations (i.e., positive freedom) (Sen 1988). In this conceptualization, freedom is more than a goal; it paves the way for development. Integrating the positive dimension of peace and freedom resonates with the wider spectrum of sustainability, from the attainment of basic needs for human survival to the promotion of individual agency, equity, and opportunity for human flourishing. The environment-peace nexus also speaks to this integration, recognizing human–nature entanglements to prompt safeguarding not only of human development but also of ecological integrity, across temporalities and territorialities.

Given the interdependencies between environmental, social, and economic objectives, balancing tradeoffs in values and outcomes is required to achieve sustainability. This implies that conflict, if left unmanaged, can frustrate progress in the sustainability agenda, and it follows that the pursuit of peace may be symbiotic to it. As our understanding of the interdependencies between social, environmental, and economic components has evolved over time, researchers and policy makers increasingly acknowledge the complex and multidimensional character of the sustainability agenda (Alkire and Santos 2010, 2014; Mayer 2008). Often, different goals are pursued within different governance models varying according to legal frameworks, decision-making capacities, connectivity, and knowledge at national and local scales (Morita et al. 2019).

Overall, sustainability initiatives tend to downplay the potential conflict or tensions arising from incompatible goals and objectives or power imbalances. To this end, Nilsson et al. (2016) explicitly provide a framework for evaluating sustainability goals and interactions to encourage integrated decision-making towards positive change. Other integrative modeling techniques attempt to understand causal links between goals, policies, and related interdependencies (Collste et al. 2017). However, such models and frameworks explicitly evaluate specific goals and policies, rather than outlining the broader dynamics that drive or constrain their attainment. More robust modeling is needed to demonstrate the (bi)directional causality between sustainability goals and conditions of peace/conflict under various socio-political–ecological contexts.

The loci of decision-making (e.g., governance frameworks and institutions) can remain inflexible to the complex and evolving requirements of the sustainability agenda. Dryzek and Pickering (2019) argue that established practices and institutions purportedly promoting sustainability have become too static and co-opted, instead of being capable of self-scrutiny and change, thereby reinforcing and perpetuating ecologically harmful ideas and practices. When questions of peace and sustainability confront social, environmental, and economic trade-offs, it is these processes of decision-making and the implicit hierarchies created through them that require further scrutiny. We propose that empirical modeling can assist policy makers in understanding the enabling conditions and mechanisms which contribute to achievement of integrated sustainability, and allow for policy development that is better tailored to the individual nuances of various socio-political–ecological contexts, thus better supporting the sustainability agenda overall (Galdeano-Gómez et al. 2016). Nevertheless, modeling alone is insufficient to guide policy development because models are built around assumptions and uncertainties. However, they can illuminate trade-offs and scenarios to better inform policy design and implementation (Saltelli et al. 2020).

### Formalizing a framework for integrated sustainability

We present a framework for integrated sustainability based on four propositions synthesized from the discussion above. The key components of the framework are based around the following: (1) the ability of humans to meet their basic needs; (2) the importance of having a large range of choices to meet their potential; and (3) issues of generational equity implicit (and at times explicit) in our understanding of well-being, all within the constraints of current environmental capacity. These are encapsulated in the following propositions.

Proposition one: Sustainable development involves the prevention of deprivation in basic human needs

Proposition two: Sustainable development involves the promotion of individual agency, equity, and opportunity to define and pursue subjective values

Proposition three: Sustainable development involves the safeguarding of public, social, and environmental goods across temporally and spatially nested social-ecological systems

The first of these propositions deals explicitly with the provision of physical, environmental and social goods, services and structures needed for human security, identity, and physical well-being. Building on this, the second proposition suggests that the sustainability agenda involves the expansion of individual freedoms and opportunities to pursue or expand subjective utility, following concepts of positive peace and freedom, and well-being. It bears mentioning here that “individual agency” in Proposition 2 refers to agency of the appropriate decision-making unit, where the decision-making unit can be an individual, community, or any collective/group that may, from time to time, decide as a single entity. The third proposition involves temporal, spatial, and ecological considerations. Collectively, these three propositions constitute the generally accepted conceptualizations of the sustainability agenda, but do not go so far as to specify how to achieve it. As discussed earlier, the pursuit of sustainable development is fraught with arguments over tradeoffs. For instance, there are debates on the “substitutability” of certain human and environmental resources towards economic development ends (i.e., Heal 2012). However, an integrated conceptualization of sustainability suggests that not all human–nature dependencies are substitutable or counter-balanced by developing other forms of capital—in effect, some tradeoffs may threaten the overall viability of the social-ecological system itself (Rockström et al. 2009). The key to sustainable development then is balancing the tradeoffs created at the human decision-making level that are inherent in the sustainability agenda. This requires a fourth proposition.

Proposition four: Sustainable development involves the process of resolving the inherent incompatibilities between human development and ecological integrity through institutions that facilitate cooperation and regulate competition in social-ecological systems

This fourth proposition suggests that managing conflicts inherent in the sustainability agenda is best performed by institutions that facilitate collaboration across spatially, temporally, and socially nested sub-systems (Ostrom 2005). Here institutions are understood as “…stable, valued recurring patterns of behavior” (Huntington 1968, p 12), and can be either formal as inscribed in the laws and rules established to govern a society, or informal as in the cultural values and norms that are broadly accepted within a society or social group. Both formal and informal institutions are utilized by societies to manage conflicts, but with regards to this proposition we assert that institutions that both enable cooperation across networks and social ties as well as those that effectively regulate competition are required to pursue the sustainability agenda (Schnegg 2018). This proposition draws on a well-established body of research related to effective cooperation and conflict resolution (Coleman et al. 2017a,^ 2019^; b; Deutsch 1973, 1985, 2006; Rubin et al. 1994).

Where the 2030 Sustainability Agenda and associated SDGs tend to be output and outcome-oriented, the propositions above organize the commitments and framing of the goals into a framework that describes what the goals are meant to achieve (Propositions 1–3) and the mechanisms by that drive or influence implementation and goal attainment (Proposition 4).

### Formalizing the model

Most sustainability measures account for some interaction between basic needs deprivations, subjective well-being, environmental quality, and policy and governance. However, as of yet no proposed framework is adequately able to capture the dynamic interplay between nested subsystems across time and space, nor the capacity for institutions to resolve development conflicts and foster collaboration.

**Fig. 1:**
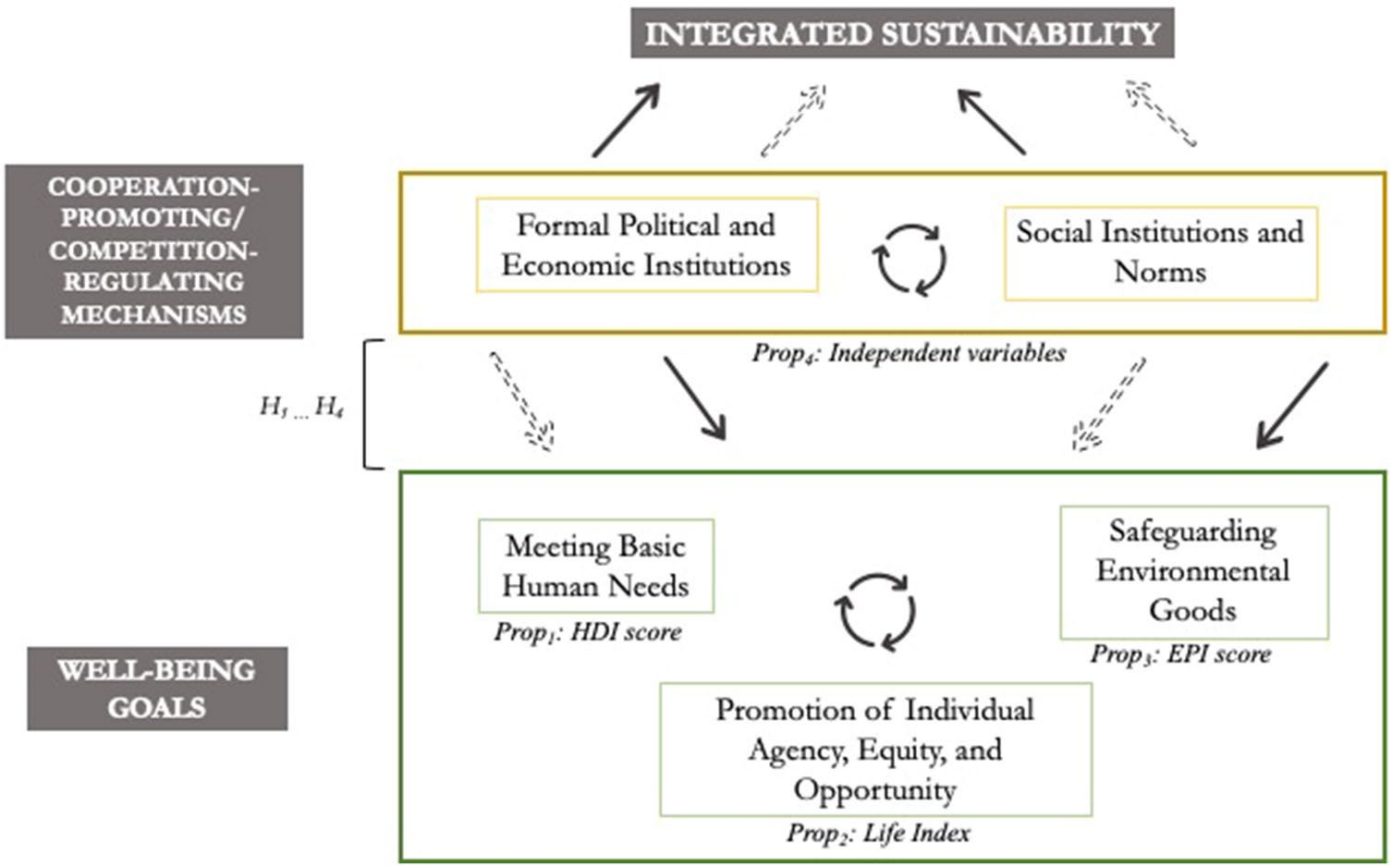
A framework for integrated sustainability

We propose that integrated sustainability is the achievement of both compatible and competing socio-economic-environmental well-being goals via a set of institutions, policies and cooperative structures that enable societies to balance the trade-offs inherent in nested social-ecological systems (Fig. 1). Integrated sustainability is met when these goals are achieved in a manner that allows for continuity.

This simplified framework describes institutions (formal and informal) as the mechanisms that enable societies to balance tradeoffs across competing goals and agendas. The question, then, is whether that pattern is borne out in the real world. In other words, do systems that enable cooperation or regulate competition across incompatible goals and objectives perform better in achieving sustainability outcomes? The next section explores this question through a series of tested hypotheses.

## Methods

We conduct an empirical analysis of the propositions outlined above by utilizing existing metrics of sustainability as outcome variables to stand as proxies for Propositions 1–3. Specifically, we use linear regression to assess the effect of a series of independent variables that represent the types of institutional mechanisms described in Proposition 4 on the sustainability outcomes in Propositions 1–3. A summary of the dependent and independent proxy variables to measure our hypotheses on integrated sustainability outcomes are presented in Table 1 and are described further below.

**Table 1:** Variables description

Variable type	Variable name	Description	Source	Original Scale	Scale changes
Dependent variables	HDI	Aggregate HDI values for country groups (by human development category, region and the like) are calculated by applying the HDI formula to the weighted group averages of component indicators. Life expectancy and GNI per capita are weighted by total population, expected years of schooling is weighted by population ages 5–24 and mean years of schooling is weighted by population ages 25 and older	United Nations Development Programme	0–1	
	EPI	EPI reveals a tension between two fundamental dimensions of sustainable development: (1) environmental health, which rises with economic growth and prosperity, and (2) ecosystem vitality, which comes under strain from industrialization and urbanization	Collaboration of the Yale Center for Environmental Law and Policy (YCELP), Yale University, Columbia University Center for International Earth Science Information Network (CIESIN), and the World Economic Forum (WEF)	0–100	0 to 1
	Life Today	Question asked: please imagine a ladder with steps numbered from 0 at the bottom to 10 at the top. Suppose we say that the top of the ladder represents the best possible life for you, and the bottom of the ladder represents the worst possible life for you. On which step of the ladder would you say you personally feel you stand at this time, assuming that the higher the step the better you feel about your life, and the lower the step the worse you feel about it? Which step comes closest to the way you feel?	Gallup World Poll	0–100	0 to 1
	Life Evaluation Index	The Life Evaluation Index measures respondents’ perceptions of where they stand now and in the future	Gallup World Poll	0–1	
Control variables	Polity revised combined score	This variable is a modified version of the Polity variable added in order to facilitate the use of the Polity regime measure in time-series analyses	Varieties of Democracy (V-Dem) Project		0 to 1
	GDP per capita	GDP per capita is a measure of a country's economic output that accounts for its number of people	World Bank	Min: 710.8; Max: 123,308.2	Take log and then change the scale to 0–1
	Income_group	The World Bank divides the world's economies into four income groups: high, upper-middle, lower-middle, and low. (World Bank)	World Bank	1–4	0–1
Independent Variables of Hypothesis 1	Equal protection index	Question asked: how equal is the protection of rights and freedoms across social groups by the state? Equal protection means that the state grants and protects rights and freedoms evenly across social groups. To achieve equal protection of rights and freedoms, the state itself must not interfere in the ability of groups to participate and it must also take action to ensure that rights and freedoms of one social group are not threatened by the actions of another group or individual	Varieties of Democracy (V-Dem) Project	0–1	
	Government effectiveness	The variable combines into a single grouping responses on the quality of public service provision, the quality of the bureaucracy, the competence of civil servants, the independence of the civil service from political pressures, and the credibility of the government’s commitment to policies. The main focus of this index is on "inputs" required for the government to be able to produce and implement good policies and deliver public goods	World Bank	− 2.5 to 2.5	0–1
Independent Variables of Hypothesis 2	Control of corruption	The variable measures perceptions of corruption, conventionally defined as the exercise of public power for private gain. The particular aspect of corruption measured by the various sources differs somewhat, ranging from the frequency of "additional payments to get things done", to the effects of corruption on the business environment, to measuring "grand corruption" in the political arena or in the tendency of elite forms to engage in "state capture"	World Bank	− 2.5 to 2.5	0 to 1
	Regulatory quality	The variable includes measures of the incidence of market-unfriendly policies such as price controls or inadequate bank supervision, as well as perceptions of the burdens imposed by excessive regulation in areas such as foreign trade and business development	World Bank	− 2.5 to 2.5	0–1
	Rule of law	The variable measures the independence of the judiciary; the extent to which rule of law prevails in civil and criminal matters; the existence of direct civil control over the police; the protection from political terror, unjustified imprisonment, exile and torture; absence of war and insurgencies; and the extent to which laws, policies and practices guarantee equal treatment of various segments of the population	World Bank	− 2.5 to 2.5	0–1
	Polity Competition	Question asked: is there any (institutionalized) political competition?	Varieties of Democracy (V-Dem) Project	1–10; − 66; − 77; − 88	0–1
Independent Variables of Hypothesis 3	Equal Distribution of Resources Index	Question asked: how equal is the distribution of resources? Clarification: this component measures the extent to which resources—both tangible and in- tangible—are distributed in society	Varieties of Democracy (V-Dem) Project	0–1	
	Voice and accountability	The variable includes a number of indicators measuring various aspects of the political process, civil liberties and political rights. These indicators measure the extent to which citizens of a country are able to participate in the selection of governments. This category also includes indicators measuring the independence of the media, which serves an important role in monitoring those in authority and holding them accountable for their actions	World Bank	− 2.5 to 2.5	0–1
	Freedom of expression and alternative sources of information index	Question asked: to what extent does government respect press and media freedom, the freedom of ordinary people to discuss political matters at home and in the public sphere, as well as the freedom of academic and cultural expression?	Varieties of Democracy (V-Dem) Project	0–1	

### Variable selection

Whereas Proposition 1 refers to the fulfillment of basic needs (or prevention of their deprivation more formally), we can assume that at the country level, achievement on the Human Development Index (HDI) should approximate a country’s success at delivering this first aspect of well-being (UNDP 2010, 2019). For a given country, let DV_1_ stand as the first parameter of integrated sustainability such that:

$${\text{Prop}}_{1} :{\text{DV}}_{1} = {\text{HDI score}}$$

Although other measures such as the Multi-Dimensional Poverty Index (Alkire and Santos 2010, 2014) might be a better theoretical indicator to measure the fulfillment of basic needs, MPI has much fewer available data than HDI and would result in a dataset with a severely limited sample of *n* = 36 countries in 2014 for MPI, and it is not sufficient to run the analysis in our study.

Whereas Proposition 2 refers to the promotion of individually defined goals and objectives, what Galtung would call somatic realization, the Gallup World Poll’s (2014) indices of thriving approximate a country’s ability to enable that higher level of well-being by measuring the degree to which citizens of the country experience actualization. We use two variables from the Gallup World Poll: Life Today and Life Evaluation Index. We average them to create our Life Index. For a given country in a given year, let DV_2_ stand as the second parameter of integrated sustainability such that:

$${\text{Prop}}_{2} :{\text{DV}}_{2} = {\text{Life Index}}\left( {\text{mean of Life Today and Life Evaluation}} \right)$$

Proposition 3 is more difficult to observe, given the intergenerational aspect of the safeguarding of social and environmental goods. However, we can assume that the degree to which a country safeguards these goods in the present will directly impact its ability to maintain them in the future. Thus, we can utilize the Environmental Performance Index (EPI) to assess that process of safeguarding environmental integrity (Wendling et al. 2018). For a given country in a given year, let DV_3_ stand as the third parameter of integrated sustainability such that

$${\text{Prop}}_{3} :{\text{DV}}_{3} = {\text{EPI score}}$$

Finally, if these three individual variables collectively constitute the integrated system, we can construct a simple composite measure of integrated sustainability by averaging the three outcome variables. For a given country in a given year, let DV_4_ stand as the measure of integrated sustainability such that

$${\text{DV}}_{4} = {\text{mean of DV}}_{{1\left( {{\text{HDI}}} \right)}} ,{\text{DV}}_{{2\left( {\text{Life Index}} \right)}} ,{\text{DV}}_{{3({\text{EPI}})}}$$

This formalization, however, does not fully capture Proposition 4 because that proposition describes the process of effectively resolving conflicts inherent in Propositions 1–3. To fully observe Proposition 4, we need to measure both the outcome variable as well as the relationships between various institutions and the observed outcome. If we select a series of proxy measures for those institutions as independent variables (IVs), we can measure that relationship of DVs to IVs using linear regression with the equation *y′* = *a* + *bx*. We can, therefore, observe Proposition 4 for a given country in a given year such that

$${\text{Prop}}_{4} :{\text{DV}}_{4} = a + bx_{i \ldots j}$$

where *x*_*i*…*j*_ represents the influence of a set of institutional IVs

In order to identify the correct set of IVs to observe Proposition 4, we hypothesize a series of relationships between institutions and the well-being components of integrated sustainability (DV_1–3_).

H_1_: Countries with institutions that enable cooperation across social groups and incompatible interests will achieve higher performance in each component of sustainability DV_1_–DV_3_.

H_2_: Countries with institutions that effectively regulate competition across social groups and incompatible interests will achieve higher performance in each component of sustainability DV_1_–DV_3_.

H_3_: Informal social and cultural institutions are primarily responsible for a country’s attainment on DV_2,_ whereas formal political and economic institutions are primarily responsible for a country’s performance on DV_1_ and DV_3_.

The most comprehensive repository of institutional indicators available is the Versions of Democracy (V-Dem) database (Coppedge et al. 2020) which contains more than 3000 unique measures of formal social, political, and economic institutions at the country level covering the years 1785–2018. We reviewed these indicators for relevant proxies of institutions that enable cooperation. The extended version of the V-Dem database includes a range of additional data including the Worldwide Governance Indicators (WGI) from the World Bank. Composite governance indicators including voice and accountability, regulatory quality, rule of law, government effectiveness, and control of corruption have previously been utilized to assess institutional influence on the EPI (Mavragani et al. 2016). We extend that approach by exploring their relationship to HDI, Life Index, and our composite for integrated sustainability.

For H_1_, we employ two proxy variables to measure countries’ institutional abilities to enable effective cooperation, including the Equal Protection Index and Government Effectiveness. For H_2_, we chose four proxy variables to measure countries’ institutional abilities to regulate competition, including control of corruption, regulatory quality, rule of law, and political competition.

As for H_3_, we anticipate that a portfolio of interconnected institutions will be required to drive sustainability attainment. Previous research has shown that environmental performance and development tend to be driven by similar institutional characteristics, and that those tend to be closely associated with formal political and economic regulatory schemas (Mukherjee and Chakraborty 2013). Social thriving, however, is a fundamentally more nebulous and subjective concept. As a result, we assume that informal institutions will play a larger role in attaining this dimension of integrated sustainability. As proxies for informal institutions, we select three indicators that most closely approximate the theorized relationships: equal distribution of resources, voice and accountability, and freedom of expression.

As proxies, the IVs used in H_1–3_ represent only a narrow set of institutions; they are inadequate to fully scrutinize Proposition 4 in the theoretical model of integrated sustainability. We assume that the effects of those narrowly measured institutions correspond to a broader, more holistic institutional effect on integrated sustainability captured in DV_4_. We can formalize this in a fourth hypothesis as follows:

H_4_: Institutions associated with promotion of cooperation and regulation of competition will have distinct effects on integrated sustainability and will correlate with discrete underlying factors.

Following Gallego-Alvarez et al. (2014), we anticipate that structural characteristics like income and political architecture will likewise impact a country’s performance on each of the aspects of sustainability outlined above. Therefore, we include log GDP, which calculates the log of GDP per capita, from the World Bank. We also include the Polity Score, which measures the polity regime from + 10 (strongly democratic) to − 10 (strongly autocratic), in the V-Dem dataset.

### Model estimation

Because of the wide array of indicators for dependent and independent variables, we face data constraints. We employ a cross-sectional approach that utilizes data for 199 countries from the year 2014, which had the most comprehensive data coverage. To enable comparisons across each IV, all data are re-scaled to range from 0 to 1.

We begin by estimating the effect of each set of institutional IVs using the equation for linear regression *y′* = *a* + *bx* for each of the individual DVs_1–3_*.* This enables us to identify the specific influence of variables that proxy formal institutional arrangements to enable cooperation and regulate competition and variables that proxy informal institutions on various sustainability outcomes. We utilize a stepwise addition approach to identify the effect of each IV on the respective DVs. GDP has been shown to have a large effect on several of our DVs that can obscure the impact of other factors. We thus begin the stepwise addition with the IVs and end with the controls.

Testing H_4_ requires additional statistical estimations to first identify the underlying factors in our data, then estimate the effect of those factors on our measure of integrated sustainability Prop_4_. A principal components analysis (PCA) (Pearson 1901) enables dimension reduction among correlated data. Similar to Mavragani et al. (2016), we undertake a PCA to determine whether there are underlying factors that can be attributed to outcomes in the integrated sustainability model. Because we are interested in understanding the role of formal and informal institutions on integrated sustainability outcomes, we estimate the PCA using only the independent variables and omitting the controls. We then use the linear regression equation presented above to estimate the effect of the resulting factors on DV_4_.

## Results

We begin by presenting the results of our statistical analyses of H_1_–H_3_. Table 2 presents regression results in step-wise order for the variables selected as proxies for formal institutions that enable cooperation (H_1_). In Model 1, the variable for Equal Protection is positively and significantly correlated with each IV*.* However, when Government Effectiveness is added in Model 2, the significance of Equal Protection drops off for HDI and EPI, and the sign flips to a significant and negative correlation for Life Index, which will be later discussed. Government Effectiveness maintains a positive and significant effect when controls are added in Model 3. In both Models 2 and 3, we have conditional evidence to support H_1_, and the *F* statistic of all models indicates that the IVs are better predictors than random variables. Overall, the results show countries with higher Government Effectiveness will achieve higher performance in HDI, Life Index and EPI.

**Table 2:** Step-wise regression of cooperative institutions on sustainability index

Dependent variable	Independent variable	Model 1	Model 2	Model 3
I1—HDI	(Intercept)	0.509***	0.386***	0.309***
	Equal protection_2014	0.291***	− 0.033	0.010
	Government effect_2014		0.644***	0.137***
	e_polity2_2014			0.052***
	logGDP_2014			0.526***
	*R*^2^	0.210	0.733	0.924
	Adjusted *R*^2^	0.205	0.730	0.922
	*F* statistics	45.169***	232.122***	465.934***
	*df*	(1170)	(2169)	(4153)
	*n*	172	172	158
I2—Life Index	(Intercept)	0.290***	0.186***	0.112***
	Equal protection_2014	0.175***	− 0.103*	− 0.090*
	Government effect_2014		0.524***	0.237**
	e_polity2_2014			0.072*
	logGDP_2014			0.315***
	*R*^2^	0.107	0.509	0.635
	Adjusted *R*^2^	0.101	0.502	0.624
	*F* statistics	17.120***	73.079***	57.813***
	*df*	(1143)	(2141)	(4133)
	*n*	145	144	138
I3—EPI	(Intercept)	0.171***	0.006	− 0.081
	Equal protection_2014	0.489***	0.029	0.084
	Government effect_2014		0.896***	0.424***
	e_polity2_2014			0.048
	logGDP_2014			0.504***
	*R*^2^	0.274	0.732	0.826
	Adjusted *R*^2^	0.270	0.729	0.821
	*F* statistics	63.087***	226.439******	180.310
	*df*	(1167)	(2166)	(4152)
	*n*	169	169	157

***p < 0.001; **p < 0.01; *p < 0.05; *p* < 0.1

Table 3 presents the results of models that explore the effect of formal institutions that regulate competition on the three sustainability outcomes (H_2_). In Models 1–4, Regulatory Quality plays a strong positive and significant role in predicting sustainability outcomes across the DVs. In contrast, Political Competition has a negative effect, but is only marginally significant for HDI. Interestingly, for outcomes in HDI, that marginal negative impact is matched with a large, positive, and significant effect of Regulatory Quality. This suggests that unchecked political competition may have a deleterious impact on sustainability outcomes. For EPI in Model 4, Regulatory Quality is marginally significant, but Rule of Law has a large, positive, significant effect. In contrast, Life Index is predicted by a combination of Regulatory Quality and Control of Corruption. Together, the results of Model 4 suggest that regulating competition is important for sustainability outcomes. Interestingly, income as measured by log GDP nullifies these dynamics.

**Table 3:** Step-wise regression of competition regulation on sustainability index

Dependent variable	Independent variable	Model 1	Model 2	Model 3	Model 4	Model 5
I1—HDI	(Intercept)	0.510***	0.450***	0.421***	0.444***	0.319***
	Control of corruption_2014	0.453	0.166	− 0.012	0.014	0.006
	Regulatory quality_2014		0.370***	0.227	0.312	0.081
	Rule of law_2014			0.336	0.252	− 0.013
	Political competition_2014				− 0.047	0.010
	e_polity2_2014					0.050
	logGDP_2014					0.577***
	*R*^2^	0.525	0.595	0.604	0.595	0.912
	Adjusted* R*^2^	0.522	0.590	0.597	0.584	0.909
	*F* statistics	187.981***	123.975***	85.476***	55.785***	253.557***
	*df*	(1170)	(2169)	(3168)	(4152)	(6146)
	*n*	172	172	172	157	153
I2—Life Index	(Intercept)	0.252***	0.224***	0.237***	0.242***	0.132***
	Control of corruption_2014	0.354***	0.228**	0.308*	0.288*	0.178
	Regulatory quality_2014		0.160	0.223	0.260*	0.071
	Rule of law_2014			− 0.150	− 0.141	− 0.149
	Political competition_2014				− 0.024	− 0.018
	e_polity2_2014					0.065
	logGDP_2014					0.379***
	*R*^2^	0.424	0.438	0.440	0.445	0.614
	Adjusted* R*^2^	0.420	0.430	0.428	0.428	0.596
	*F* statistics	104.482***	54.951***	36.709***	26.459***	33.961***
	*df*	(1142)	(2141)	(3140)	(4132)	(6128)
	*n*	144	144	144	137	135
I3—EPI	(Intercept)	0.199***	0.120***	0.071*	0.077*	− 0.065*
	Control of corruption_2014	0.699***	0.318***	0.016	− 0.032	− 0.010
	Regulatory quality_2014		0.489***	0.247	0.262	0.059
	Rule of law_2014			0.569*	0.649**	0.291
	Political competition_2014				− 0.049	0.038
	e_polity2_2014					0.040
	logGDP_2014					0.625***
	*R*^2^	0.573	0.629	0.641	0.648	0.816
	Adjusted *R*^2^	0.570	0.624	0.635	0.639	0.808
	*F* statistics	223.970***	140.595***	98.296***	71.031***	107.845***
	*df*	(1167)	(2166)	(3165)	(4154)	(6146)
	*n*	169	169	169	159	153

***p < 0.001; **p < 0.01; *p < 0.05; *p* < 0.1

Exploration of H_3_ (Tables 4 and 5) suggests that a different set of institutions are required to achieve higher life actualization than are required to advance basic needs and protect environmental integrity. Table 4 presents the results of models with proxies of informal institutions that enable cooperation and regulate competition as the IVs. Contrary to our hypothesis, informal institutions appear to have a similar effect on all three DVs. Specifically, Voice and Accountability is a strong and positive predictor for HDI, Life Index and EPI, suggesting that allowing for a plurality of views, perspectives, and agendas in the political discourse is important for sustainability. Interestingly, Freedom of Expression is a weak and negative predictor, suggesting some expression may need to be regulated to achieve better outcomes, perhaps to mitigate the deleterious impact of false information or forms of expression that intimidate or silence others.

**Table 4:** Step-wise regression of Informal institutions on sustainability index

Dependent variable	Independent variable	Model 1	Model 2	Model 3	Model 4
I1—HDI	(Intercept)	0.428***	0.398***	0.481******	0.327***
	Equal distribution_2014	0.447***	0.376***	0.281	0.095***
	Voice and accountability_2014		0.134***	0.463***	0.049
	Freedom of expression_2014			− 0.293***	− 0.073*
	e_polity2_2014				0.090***
	logGDP_2014				0.527***
	*R*^2^	0.612	0.641	0.696	0.929
	Adjusted* R*^2^	0.610	0.637	0.691	0.926
	*F* statistics	268.276***	150.946	128.206***	395.460***
	*df*	(1170)	(2169)	(3168)	(5152)
	*n*	172	172	172	158
I2—Life Index	(Intercept)	0.243***	0.198***	0.328***	0.187***
	Equal distribution_2014	0.259***	0.148***	0.021	− 0.114**
	Voice and accountability_2014		0.203***	0.645***	0.365***
	Freedom of expression_2014			− 0.415***	− 0.177*
	e_polity2_2014				− 0.010
	logGDP_2014				0.404***
	*R*^2^	0.268	0.346	0.466	0.646
	Adjusted* R*^2^	0.263	0.337	0.455	0.633
	*F* statistics	52.261***	37.345***	40.716***	48.220***
	*df*	(1143)	(2141)	(3140)	(5132)
	*n*	145	144	144	138
I3 – EPI	(Intercept)	0.111***	0.051	0.186***	− 0.001
	Equal distribution_2014	0.625***	0.477***	0.321***	0.139**
	Voice and accountability_2014		0.274***	0.780***	0.354
	Freedom of expression_2014			− 0.452***	− 0.132
	e_polity2_2014				0.013
	logGDP_2014				0.573***
	*R*^2^	0.552	0.609	0.668	0.804
	Adjusted* R*^2^	0.550	0.604	0.662	0.797
	*F* statistics	206.166***	129.081***	110.561***	123.856***
	*df*	(1167)	(2166)	(3165)	(5151)
	*n*	169	169	169	157

***p < 0.001; **p < 0.01; *p < 0.05; *p* < 0.1

**Table 5:** Step-wise regression of informal institutions on Life Index by income group

Life Index by income group	Dependent variable	Model 1	Model 2	Model 3	Model 4
Low	(Intercept)	0.295***	0.284***	0.287***	0.217**
	EqualDistribution_2014	− 0.141	− 0.156	− 0.158	− 0.121
	VoiceAndAccountability_2014		0.041	0.050	0.013
	FreedomOfExpression_2014			− 0.008	− 0.069
	e_polity2_2014				0.180*
	*R*^2^	0.115	0.079	0.034	0.192
	Adjusted* R*^2^	0.153	0.160	0.160	0.333
	*F* statistics	3.985	1.993	1.267	2.367
	*Df*	(122)	(221)	(320)	(419)
	*N*	24	24	24	24
Lower middle	(Intercept)	0.341***	0.323******	0.342***	0.3***43
	EqualDistribution_2014	− 0.023	− 0.035	− 0.054	− 0.032
	VoiceAndAccountability_2014		0.053	0.202	− 0.138
	FreedomOfExpression_2014			− 0.115	− 0.157
	e_polity2_2014				0.255*
	*R*^2^	− 0.027	− 0.050	− 0.065	0.074
	Adjusted* R*^2^	0.004	0.014	0.032	0.189
	*F* statistics	0.143	0.216	0.332	1.635
	*Df*	(132)	(231)	(330)	(428)
	*N*	34	34	34	33
Upper middle	(Intercept)	0.391***	0.357***	0.388***	0.390***
	EqualDistribution_2014	0.027	0.008	− 0.013	− 0.020
	VoiceAndAccountability_2014		0.088	0.297	0.398
	FreedomOfExpression_2014			− 0.188	− 0.128
	e_polity2_2014				− 0.122
	*R*^2^	− 0.025	− 0.026	− 0.006	− 0.023
	Adjusted* R*^2^	0.003	0.029	0.076	0.090
	*F* statistics	0.100	0.523	0.932	0.795
	*Df*	(136)	(235)	(334)	(432)
	*N*	38	38	38	37
Upper	(Intercept)	0.525***	0.505***	0.634***	0.768***
	EqualDistribution_2014	0.003	− 0.121	− 0.284*	− 0.499***
	VoiceAndAccountability_2014		0.169	0.890***	1.109***
	FreedomOfExpression_2014			-0.651***	− 0.326*
	e_polity2_2014				− 0.441***
	*R*^2^	− 0.022	0.065	0.341	0.509
	Adjusted* R*^2^	0.000	0.105	0.383	0.552
	*F* statistics	0.000	2.628	9.095***	12.640***
	*Df*	(146)	(245)	(344)	(441)
	*N*	48	48	48	46

***p < 0.001, **p < 0.01, *p < 0.05, *p* < 0.1

Table 4 also highlights another pattern: when control variables are added in Model 4, Equal Distribution of Resources flips from a null effect in Model 3 to a negative and significant effect for Life Index only. Thus, there may be some indirect evidence for H_3_. To further explore this finding, we reran the regressions separately for each of the World Bank (2020) income groupings. The results presented in Model 4 in Table 5 show that for low and lower-middle income countries, political architecture as measured by the polity score is the key predictor of the Life Index, where more democratic countries achieve better outcomes. In contrast, in high-income countries, Equal Distribution, Freedom of Expression, and more democratic institutions are all significant negative predictors of Life Index. It is possible that institutional dynamics of higher income countries may be exerting undue impact in our model. Another possibility is that quality of life issues are perhaps more closely linked with income expectations in lower and middle-income countries.

**Fig. 2:**
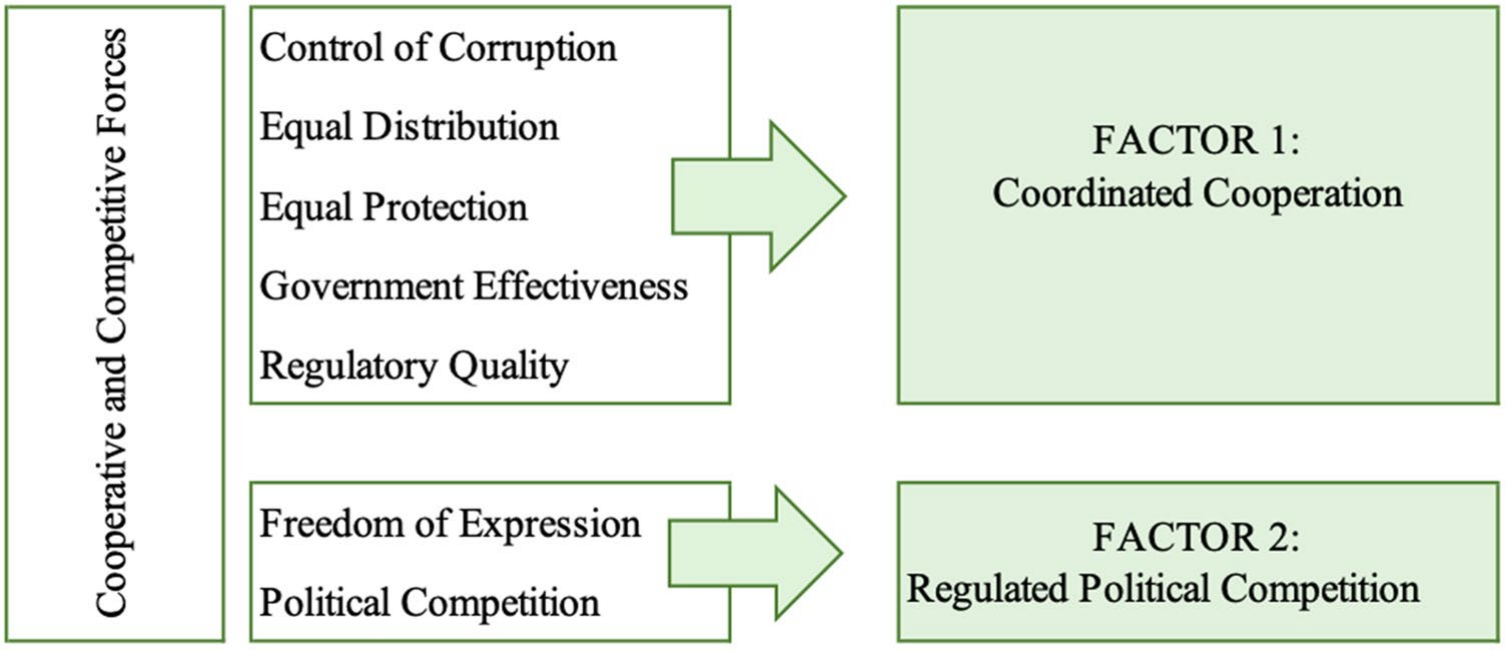
Factors obtained from a PCA of the 9 IVs

From the preceding models, it is clear that we are only measuring narrow portions of incredibly complex institutional dynamics in each of the three DVs. Moreover, in isolation, each DV captures only a narrow aspect of integrated sustainability. We, therefore, generated a composite index of integrated sustainability and conducted Principal Components Analysis (PCA) of the nine IVs to assess whether there are underlying factors that might better predict the integrated measure. Figure 2 presents the results of the PCA model. Our nine predictor variables loaded onto two common factors. Based on the variable groupings, we termed the following factors: (1) coordinated cooperation and (2) regulated political competition. The two extracted factors cumulatively explain 87.4% of the variance in the data.

The two derived factors were then used as IVs to predict the combined index DV_4_. We limited the use of the results of the PCA only to the integrated model to avoid over-specification and false precision in our analysis. As shown in Table 6, Factor 1 (Coordinated Cooperation) is a strong and positive predictor of the integrated sustainability index across all models. Factor 2 (Regulated Political Competition) is a weaker but still significant negative predictor of integrated sustainability, suggesting that too much competition may be detrimental to integrated sustainability. Together, our findings align with the theoretical model in Fig. 1 and also provide conditional support for H_4_. In keeping with prior findings, introducing controls reduces the effect of Factors 1 and 2.

**Table 6:** Step-wise regression of integrated factors on Sustainability Index

Dependent variable	Independent variable	Model 1	Model 2	Model 3
I4—Integrated Index	(Intercept)	0.196***	0.247***	0.138***
	factor1	0.635***	0.732***	0.230***
	factor2		− 0.145***	− 0.111*
	e_polity2_2014			0.123**
	logGDP_2014			0.502***
	*R*^2^	0.679	0.705	0.902
	Adjusted* R*^2^	0.677	0.701	0.899
	*F* statistics	290.017***	157.761***	299.071***
	*df*	(1137)	(2132)	(4130)
	*N*	139	135	135

***p < 0.001; **p < 0.01; *p < 0.05; *p* < 0.1

In order to explore the effect of log GDP, we again stratified the sample into World Bank income categories and reran the regressions for each grouping. As shown in Table 7, while regulation of political competition may inhibit the achievement of integrated sustainability in lower-middle and upper-middle income countries, it is coordinated cooperation that positively predicts the integrated sustainability index in higher income countries.

**Table 7:** Step-wise regression of integrated factors on Sustaibility Index by income group

Integrated Index by income group	Dependent variable	Model 1	Model 2	Model 3
Low	(Intercept)	0.278***	0.349***	0.332***
	factor1	0.089	0.081	0.106
	factor2		− 0.100	− 0.184
	e_polity2_2014			0.100
	*R*^2^	− 0.016	0.006	0.018
	Adjusted* R*^2^	0.032	0.105	0.166
	*F* statistics	0.662	1.056	1.124
	*df*	(120)	(218)	(317)
	*n*	22	21	21
Lower middle	(Intercept)	0.345***	0.369***	0.379***
	factor1	0.231	0.244	0.228
	factor2		− 0.042	− 0.405**
	e_polity2_2014			0.345**
	*R*^2^	0.102	0.046	0.239
	Adjusted* R*^2^	0.130	0.107	0.312
	*F* statistics	4.628*	1.742	4.242*
	*df*	(131)	(229)	(328)
	*n*	33	32	32
Upper middle	(Intercept)	0.409***	0.417***	0.422***
	factor1	0.283**	0.403***	0.383***
	factor2		− 0.099*	− 0.180
	e_polity2_2014			0.084
	*R*^2^	0.220	0.300	0.295
	Adjusted* R*^2^	0.241	0.339	0.354
	*F* statistics	11.446**	8.711***	6.027**
	*df*	(136)	(234)	(333)
	*n*	38	37	37
Upper	(Intercept)	0.445***	0.445***	0.445***
	factor1	0.361***	0.434***	0.435***
	factor2		-0.067	− 0.113
	e_polity2_2014			0.044
	*R*^2^	0.606	0.647	0.640
	Adjusted* R*^2^	0.615	0.663	0.665
	*F* statistics	70.194***	41.347***	27.069******
	*df*	(144)	(242)	(341)
	*n*	46	45	4

***p < 0.001, **p < 0.01, *p < 0.05, *p* < 0.1

## Discussion

Both the theoretical model and the statistical analysis of our study attempt to illuminate how sustainability outcomes are achieved. We initially proposed that institutions which enable cooperation, or regulate and coordinate competition, enable countries to more effectively navigate inevitable conflicts between different economic, political, social, and environmental agendas. To test these propositions, we constructed a rudimentary composite index of integrated sustainability and analyzed country-level performance against a variety of formal and informal institutional proxies. Our results demonstrate that countries with institutions that enable cooperation are more likely to prevent deprivation of basic human needs (in HDI), promote individual actualization (in Life Today averaged with Life Evaluation), and safeguard environmental goods (in EPI). This result holds for the composite measure of integrated sustainability, suggesting that cooperation is critical to the achievement of sustainability.

An analysis of competition, on the other hand, does not offer such clear results—institutions regulating competition only marginally impact drivers of thriving and actualization, as well as basic and environmental needs. This may be because our variables do not truly capture the full essence of competition and are instead focused on a narrow set of political and social institutional dynamics tailored toward political competition and polarization. Specifically, while regulatory quality addresses the freedom to compete in markets, the remaining variables primarily assess political and legal freedoms. They do not fully account for the impact of competition on pro-environmental choices, technological advances, and social quality of life. While the statistical design we employed here certainly omits potentially important social, ecological, and technological competition dynamics, our results show that polarization can produce certain pathologies; the data demonstrate that the negative outcomes result from focusing on ideological dominance rather than on cooperative policies.

Global income inequality across time and space is also important to consider on the outcomes of competition variables. When stratifying data by income categories, results demonstrate that competition can have a potentially beneficial impact on sustainability outcomes in lower-middle and upper-middle income countries. This is in line with recent research that suggests the relationship between competition and inequality may depend on a country’s level of development (Zac et al. 2020). Although intra-national inequality dynamics are not considered and further disaggregation of outcomes is outside the scope of this paper, we note that prior studies have contributed to understanding these relationships between inequality and well-being. For instance, Wilkinson and Pickett (2009) use separate, extensive social indicators and find a strong correlation between income growth and enhanced quality of life in lower-income countries. In higher-income countries, however, lower levels of inequality improve quality of life, independent of income growth. Evidently, more nuanced policy and institution building is required, accounting for competition within societal and civic structures. Still, our results attest to the limits of competition in capitalist systems and suggest competitive logics such as self-maximization can limit intergenerationally-oriented behaviors and undermine integrated sustainability compared to prosocial behavior (Shahrier et al. 2017). While competition has been a central tenet in dominant neoliberal worldviews with ramifications on policy decisions, our results suggest that unfettered competition may reduce integrated sustainability.

When analyzing institutional drivers of HDI and EPI attainment, Government Effectiveness, Regulatory Quality, and Equal Distribution appear to significantly influence the achievement of higher levels of both indices. These results suggest that efficiently functioning governments and regulated markets that distribute resources equally are critical to providing an environment where basic human needs and ecological integrity are met.

Overall, the results from our hypothesis-testing suggest that there is preliminary evidence to support the assertions of the theoretical model of integrated sustainability (Fig. 1), namely that collaborative institutions are important for enabling effective management of sustainability conflicts. This idea is enshrined in SDG 16 (Peace, Justice and Strong Institutions) and SDG 17 (Partnerships for the Goals), yet many actors in the international system appear to be gravitating toward unilateral and neoliberal approaches that may not be consistent with broad cooperation and collaborative approaches. This emphasizes two concerns previously raised in this paper. First, in approaching each SDG separately, the integrative value of certain SDGs may be inadvertently discounted (Collste et al. 2017), as may be the case for SDG 16 and 17. Second, metrics used in modeling are frequently approached through a normalized neoliberal economic lens. These metrics, which are desirable goals, ground policies implemented to achieve them in similar politics. The resulting single-minded goal focus discounts process elements (Nagle et al. 2020) that are essential for encouraging interactivity and cooperation (Zhuang 2020), and creating cumulative silo-free knowledge (Ellmers 2020).

We do not suggest that either the dependent or independent variables in our study are perfect measures for integrated sustainability. Rather, we argue that the statistical relationships we report provide preliminary insight into some of the mechanisms that enable achievement of greater sustainability outcomes. Specifically, our results suggest that policy-making benefits from what Kanie et al. (2019) call ‘action coherence’ at multiple levels and across multiple sustainability goals (social, economic, political, environmental). Action coherence requires institutions that enable information flow and translation of optimization criteria across compound and concurrent decision-making processes and across social-political–ecological scales. However, many existing governance architectures treat interconnected sub-systems as discrete sector- or issue-based silos, perpetuating competition for resources, public and policymaker support, and continued stakeholder buy-in across a policy’s lifespan. While neoliberal proponents suggest that market-based competition allows for the most efficient or more effective ideas to persist, they fail to account for the multiple scales and multiple criteria for which policies must optimize. These competitive rationales which currently dominate the United States and many other systems have treated conflicts in decision-making towards sustainability as a one-off transactional approach; for instance, balancing economic growth and a transition to renewable energy. However, in reality they are more akin to multi-iteration cooperation games with evolving uncertainties and decision calculus.

While the theoretical model of integrated sustainability advanced here is rudimentary and our statistical approach far from comprehensive, we argue that a process-oriented approach to understanding the drivers of integrated sustainability can offer significant dividends for countries in achieving sustainability outcomes. Where Kanie et al. (2019) advocate more cross-silo and deliberative governance systems, our results suggest that we must understand and utilize a range of formal and informal institutions in order to establish aspirational integrated sustainability goals and coordinate across social, political and economic agendas to enable cooperative policy making and implementation. Institutional designs that acknowledge mutual interdependence and understand that choices can result in complex feedback loops and nonlinear outcomes at multiple timescales are best situated to enable integrated sustainability.

## Conclusion

The world suddenly finds itself at an important crossroad where the COVID-19 pandemic has illuminated the multi-scalar dynamics and feedback processes we discuss above. The dilemma in enacting policies to either safeguard public health or maintain economic integrity is archetypical of the types of inherent conflicts that societies face across the 2030 Sustainability Agenda. However, where those conflicts tend to be somewhat nebulous and the effects of any policy tend to be diffused, the current crisis and response policies have tangible, immediate and long-term implications. Moreover, the cross-scalar impacts and feedback processes across social, political, economic and environmental systems are undeniable, even if complex and poorly understood. Where the best public health information and good practices in disaster response suggest that coordination and cooperation are essential to effectively preventing further spread of the corona virus and preventing widespread economic distress, we are witnessing the variety of responses across different political entities, with some trending toward more cooperative approaches and others toward competition. We are thus, fortunately or unfortunately, poised for a natural experiment that will test the model of integrated sustainability we advance here in real-time.

As the world looks toward building back from the unprecedented disruptions to virtually every social, political, economic, and environmental system on the planet resulting from the pandemic, we have an opportunity to not recreate historic pathologies, but instead build back differently. Research is already beginning to show that the pandemic crisis was exacerbated in many of the hardest hit regions by a lack of cooperation, and excessive political and economic competition. Research is also beginning to demonstrate that certain populations are more vulnerable to the virus due to a host of environmentally and economically related pre-dispositions that have been central to many of the conflicts inherent in the sustainability agenda. It is therefore critically important to understand the central role that cooperation and regulated competition play in resolving those conflicts and building the institutional architecture to enable more integrated decision-making and policy.

Toward that end, we developed and tested four propositions that collectively describe the mechanisms that enable integrated sustainability. The results of our analysis suggest that institutions that enable cooperation and regulate competition are crucial in achieving sustainability outcomes. Based on our findings, we suggest that future research should more fully explore and identify the enabling conditions and mechanisms that enable attainment of better social and environmental outcomes. This, we argue, will assist policy makers and researchers pursue the 2030 Sustainability Agenda more effectively and tailor it to the individual nuances of various socio-political–ecological contexts.

## Supplementary Information

Below is the link to the electronic supplementary material.

Supplementary file1 (DOCX 23 KB)

Supplementary file2 (XLSX 10 KB)

Supplementary file3 (PDF 155 KB)

Supplementary file4 (PDF 255 KB)
