# Supplementary material for: Four propositions on integrated sustainability: toward a theoretical framework to understand the environment, peace, and sustainability nexus
Source: Sustain Sci. 2021 Mar 10;16(4):1125–45. doi: 10.1007/s11625-021-00925-y (PMC7943412; doi:10.1007/s11625-021-00925-y)
Supplement: Supplementary file 1 — Supplementary file1 (DOCX 23 KB) [file 11625_2021_925_MOESM1_ESM.docx]

**Supplementary Material 1: Codebook**

1. Dependent Variables

Variable name: **HDI**

*Human development index.* Aggregate HDI values for country groups (by human development category, region and the like) are calculated by applying the HDI formula to the weighted group averages of component indicators. Life expectancy and GNI per capita are weighted by total population, expected years of schooling is weighted by population ages 5–24 and mean years of schooling is weighted by population ages 25 and older.

Source: [UNDP 2019](http://hdr.undp.org/en/content/human-development-index-hdi)

Original Scale: 0 to 1

Scale Changes: N/A

Variable name: **EPI**

*Environmental performance index.* EPI reveals a tension between two fundamental dimensions of sustainable development: (1) environmental health, which rises with economic growth and prosperity, and (2) ecosystem vitality, which comes under strain from industrialization and urbanization.

Source: [Wendling et al 2018](https://epi.yale.edu/downloads/epi2018reportv06191901.pdf)

Original Scale: 0 to 100

Scale Changes: 0 to 1

Variable name: **Life Today**

*Life today.* Question asked: Please imagine a ladder with steps numbered from 0 at the bottom to 10 at the top. Suppose we say that the top of the ladder represents the best possible life for you, and the bottom of the ladder represents the worst possible life for you. On which step of the ladder would you say you personally feel you stand at this time, assuming that the higher the step the better you feel about your life, and the lower the step the worse you feel about it? Which step comes closest to the way you feel?

Source: [Gallup World Poll 2014](https://www.gallup.com/analytics/232838/world-poll.aspx)

Original Scale: 0 to 100

Scale Changes: 0 to 1

Variable name: **Life Evaluation Index**

*Life evaluation index.* The Life Evaluation Index measures respondents’ perceptions of where they stand now and in the future.

Source: [Gallup World Poll 2014](https://www.gallup.com/analytics/232838/world-poll.aspx)

Original Scale: 0 to 1

Scale Changes: N/A

1. Control Variables

Variable name: **Polity**

*Polity revised combined score.* This variable is a modified version of the Polity variable added in order to facilitate the use of the Polity regime measure in time-series analyses

Source: [Cooperidge et al 2020](https://www.v-dem.net/en/)

Original Scale: -10 to 10; -66; -77; -88

Scale Changes: 0 to 1

Variable name: **logGDP**

*GDP per capita.* GDP per capita is a measure of a country's economic output that accounts for its number of people.

Source: [Cooperidge et al 2020](https://databank.worldbank.org/reports.aspx?source=2&series=NY.GDP.MKTP.CD&country=)

Original Scale: 710.8 to 123308.2

Scale Changes: 0 to 1

Variable name: **Income Group**

*Income group.* The World Bank divides the world's economies into four income groups: high, upper-middle, lower-middle, and low.

Source: [Cooperidge et al 2020](https://datahelpdesk.worldbank.org/knowledgebase/articles/906519-world-bank-country-and-lending-groups)

Original Scale: 1 to 4

Scale Changes: 0 to 1

1. Independent Variables of Hypothesis 1

Variable name: **Equal Protection**

*Equal protection index.* Question asked: How equal is the protection of rights and freedoms across social groups by the state? Equal protection means that the state grants and protects rights and freedoms evenly across social groups. To achieve equal protection of rights and freedoms, the state itself must not interfere in the ability of groups to participate and it must also take action to ensure that rights and freedoms of one social group are not threatened by the actions of another group or individual.

Source: [Cooperidge et al 2020](https://www.v-dem.net/en/)

Original Scale: 0 to 1

Scale Changes: N/A

Variable name: **Government Effectiveness**

*Government effectiveness.* The variable combines into a single grouping responses on the quality of public service provision, the quality of the bureaucracy, the competence of civil servants, the independence of the civil service from political pressures, and the credibility of the government’s commitment to policies. The main focus of this index is on "inputs" required for the government to be able to produce and implement good policies and deliver public goods.

Source: [Cooperidge et al 2020](http://www.govindicators.org/)

Original Scale: -2.5 to 2.5

Scale Changes: 0 to 1

1. Independent Variables of Hypothesis 2

Variable name: **Control of Corruption**

*Control of corruption.* The variable measures perceptions of corruption, conventionally defined as the exercise of public power for private gain. The particular aspect of corruption measured by the various sources differs somewhat, ranging from the frequency of "additional payments to get things done", to the effects of corruption on the business environment, to measuring "grand corruption" in the political arena or in the tendency of elite forms to engage in "state capture".

Source: [Cooperidge et al 2020](http://www.govindicators.org/)

Original Scale: -2.5 to 2.5

Scale Changes: 0 to 1

Variable name: **Regulatory Quality**

*Regulatory quality.* The variable includes measures of the incidence of market unfriendly policies such as price controls or inadequate bank supervision, as well as perceptions of the burdens imposed by excessive regulation in areas such as foreign trade and business development.

Source: [Cooperidge et al 2020](http://www.govindicators.org/)

Original Scale: -2.5 to 2.5

Scale Changes: 0 to 1

Variable name: **Rule of Law**

*Rule of law.* The variable measures the independence of the judiciary; the extent to which rule of law prevails in civil and criminal matters; the existence of direct civil control over the police; the protection from political terror, unjustified imprisonment, exile and torture; absence of war and insurgencies; and the extent to which laws, policies and practices guarantee equal treatment of various segments of the population.

Source: [Cooperidge et al 2020](http://www.govindicators.org/)

Original Scale: -2.5 to 2.5

Scale Changes: 0 to 1

Variable name: **Political Competition**

*Political competition.* Question asked: Is there any (institutionalized) political competition?

Source: [Cooperidge et al 2020](https://www.v-dem.net/en/)

Original Scale: 1 to 10; -66; -77; -88

Scale Changes: 0 to 1

1. Independent Variables of Hypothesis 3

Variable name: **Equal Distribution**

*Equal distribution of resources index.* Question asked: How equal is the distribution of resources? Clarification: This component measures the extent to which resources — both tangible and in- tangible — are distributed in society.

Source: [Cooperidge et al 2020](https://www.v-dem.net/en/)

Original Scale: 0 to 1

Scale Changes: N/A

Variable name: **Voice and Accountability**

*Voice and accountability.* The variable includes a number of indicators measuring various aspects of the political process, civil liberties and political rights. These indicators measure the extent to which citizens of a country are able to participate in the selection of governments. This category also includes indicators measuring the independence of the media, which serves an important role in monitoring those in authority and holding them accountable for their actions.

Source: [Cooperidge et al 2020](http://www.govindicators.org/)

Original Scale: -2.5 to 2.5

Scale Changes: 0 to 1

Variable name: **Freedom of Expression**

*Freedom of expression and alternative sources of information index.* Question asked: To what extent does government respect press and media freedom, the freedom of ordinary people to discuss political matters at home and in the public sphere, as well as the freedom of academic and cultural expression?

Source: [Cooperidge et al 2020](https://www.v-dem.net/en/)

Original Scale: 0 to 1

Scale Changes: N/A

Data and replication codes available upon request.
