# Supplementary material for: Four propositions on integrated sustainability: toward a theoretical framework to understand the environment, peace, and sustainability nexus
Source: Sustain Sci. 2021 Mar 10;16(4):1125–45. doi: 10.1007/s11625-021-00925-y (PMC7943412; doi:10.1007/s11625-021-00925-y)
Supplement: Supplementary file 3 — Supplementary file3 (PDF 155 KB) [file 11625_2021_925_MOESM3_ESM.pdf]

Supplemental Material 3

Adjusted R^2 Matrix Table: H_1-H_3 Against $Prop_1$-$Prop_3$

	H_1 : Countries with institutions that enable cooperation across social groups and incompatible interests will achieve higher performance in each component of sustainability DV_1 – DV_3.		*H_2 : Countries with institutions that effectively regulate competition across social groups and incompatible interests will achieve higher performance in each component of sustainability DV_1 – DV_3.*		*H_3 : Informal social and cultural institutions are primarily responsible for a country's attainment on DV_2, whereas formal political and economic institutions are primarily responsible for a country's performance on DV_1 and DV_3.*	
	<u>Independent Variables</u>:<br>Equal Protection<br>Govt. Effectiveness		<u>Independent Variables</u>:<br>Control of Corruption<br>Regulatory Quality<br>Rule of Law<br>Political Competition		<u>Independent Variables</u>:<br>Equal Distribution of Resources Index<br>Voice and Accountability<br>Freedom of Expression & Alt. Sources of Info	
	<u>Adjusted R^2</u>:		<u>Adjusted R^2</u>:		<u>Adjusted R^2</u>:	
$Prop_1$: DV_1 = HDI score<br>Provision of Needs	0.730	0.922*	0.584	0.909*	0.691	0.926*
$Prop_2$: DV_2 = Life Index<br>Promotion of Actualization	0.502	0.624*	0.428	0.596*	0.455	0.633*
$Prop_3$: DV_3 = EPI score<br>Safeguarding Social & Ecological Goods	0.729	0.821*	0.639	0.808*	0.662	0.797*
*Adjusted R^2 with added control variables: Polity2 Score and Log GDP.						
